# Effects of taurine on male reproduction in rats of different ages

**DOI:** 10.1186/1423-0127-17-S1-S9

**Published:** 2010-08-24

**Authors:** Jiancheng Yang, Gaofeng Wu, Ying Feng, Qiufeng Lv, Shumei Lin, Jianmin Hu

**Affiliations:** 1 College of Animal Science & Veterinary Medicine, Shenyang Agricultural University, Shenyang, 110866, P.R. China

## Abstract

**Background:**

It has been demonstrated that taurine is one of the most abundant free amino acids in the male reproductive system, and can be biosynthesized by male reproductive organs. But the effect of taurine on male reproduction is poorly understood.

**Methods:**

Taurine and β-alanine (taurine transport inhibitor) were offered in water to male rats of different ages. The effects of taurine on reproductive hormones, testis marker enzymes, antioxidative ability and sperm quality were investigated.

**Results:**

The levels of T and LH were obviously increased by taurine supplementation in rats of different ages, and the level of E was also significantly elevated in baby rats. The levels of SOD, ACP, SDH and NOS were obviously increased by taurine administration in adult rats, but the levels of AKP, AST, ALT and NO were significantly decreased. The levels of SOD, ACP, LDH, SDH, NOS, NO and GSH were significantly elevated by taurine administration in aged rats, but the levels of AST and ALT were significantly decreased. The motility of spermatozoa was obviously increased by taurine supplement in adult rats. The numbers and motility of spermatozoa, the rate of live spermatozoa were significantly increased by taurine supplement in aged rats.

**Conclusions:**

The present study demonstrated that a taurine supplement could stimulate the secretion of LH and T, increase the levels of testicular marker enzymes, elevate testicular antioxidation and improve sperm quality. The results imply that taurine plays important roles in male reproduction especially in aged animals.

## Introduction

Taurine, 2-aminoethane sulphonic acid, is one of the most abundant low-molecular-weight organic constituents in human and many animals. Taurine is not involved in protein synthesis or in metabolic pathway. However, several physiological functions of taurine have been demonstrated, such as osmoregulation, calcium modulation, membrane stabilization, antioxidation, radioprotection, energy storage, xenobiotic conjugation, isethionic acid and anion balance et al. [1].

As a semi-essential amino acid, taurine is rich in many tissues, and can be biosynthesized by many tissues, such as the central nervous system [2], liver [3], kidney [4], retina [5] and mammary gland [6]. In the male reproductive system, taurine has been detected in Leydig cells, vascular endothelial cells, and some other interstitial cells of testis and epithelial cells of efferent ducts in rats [7]. It has also been reported that taurine can be biosynthesized by male reproductive organs [8]. In addition, taurine has been identified as the major free amino acid of sperm cells and seminal fluid [9-12]. Taurine may act as an antioxidant [13], capacitating agent [14,15], membrane-stabilized factor [16] and motility factor [17] of sperm. Our laboratory had previously reported that taurine can stimulate testosterone secretion in vivo and in vitro [18]. Despite its importance, the effect of taurine on the male reproduction is still unclear. The primary aim of the present study was to further investigate the effect of taurine on male reproduction in rats.

It is well known that aging results in a significant decline in male reproduction. However, a number of studies have reported that the concentration of taurine is obviously decreased in serum and some tissues of aged animals [19-21]. The correlation between the decline of taurine content and aged male reproduction was also evaluated in the present study.

## Methods

### Experimental animals and treatments

All Wistar rats were obtained from the central animal house of the Chinese Medical University. Male rats were 10 (adult) and 72 (aged) weeks old, female rats were 10 weeks old (about 200 g). They were maintained in controlled light (14h-light, 10h-dark) and temperature (22±2°C), and were given free access to rat chow and water. After acclimatizing for 1 week, all rats were used in the experiment.

Female rats were put together with male rats in cages after the oestrus of female rats were determined by vaginal smear. Impregnation was determined if sperm were detected on the second day. 15 pregnant rats with the similar expected confinement date were divided into three groups randomly, five in each group. Female rats were given different water from the day of confinement which was defined as the first day of the newborn rats. Rats in the control group were given tap water, the β-alanine group was given water containing 1%β-alanine, and the taurine group was given water containing 1% taurine. 30 male rats aged 10 weeks or 72 weeks were dived into 3 groups respectively, 10 in each group. Rats were given different treatment as female rats respectively. The study protocol was approved by our Ethical Committee and conducted in compliance to the Helsinki Declaration.

### Chemicals

Taurine and β-alanine(β-Ala) were purchased from Sigma (USA). Follicle-stimulating hormone (FSH), luteinizing hormone (LH), testosterone (T) and estradiol (E) radioimmunoassay kits were purchased from Beijing Chemclin Biotech Co., Ltd. (CHINA). Reagent kits of alkaline phosphatase (AKP), acid phosphatase (ACP), lactate dehydrogenase (LDH), superoxide dismutase (SOD), reduced glutathione hormone (GSH), malondialdehyde (MDA), sorbitol dehydrogenase (SDH), alanine aminotransferase (ALT), aspartate aminotransferase (AST), nitric oxide (NO) and nitric oxide synthase (NOS) were purchased from Nanjing Jiancheng Bioengineering Institute (CHINA).

### Sample collection and biochemical analysis

Blood was collected on the 22^nd^ day after male baby rats were killed. Adult and aged rats were killed after being treatment for 30 days, and blood, right testes and epididymides were collected. Blood was used for reproductive hormone analysis by radio immunoassay according to the introduction of reagent kits. Testes were homogenated in cold physiological saline, then were used for testis biochemical analysis by colorimetry based on the introductions of reagent kits. Epididymides were used for semen quality analysis according to the reported methods [22,23].

### Statistic analysis

Data were presented as the mean ± SE and significant differences were determined by Duncan’s multiple range test using SPSS 16.0 statistical analysis software. P values less than 0.05 were considered significant.

## Results

### Reproductive hormone analysis

**Figure 1:**
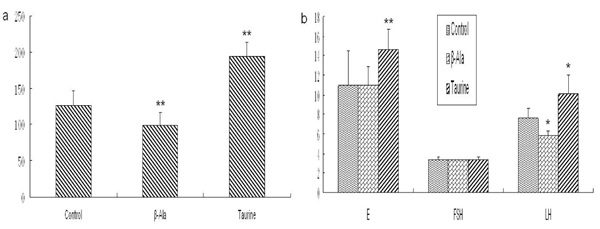
**The levels of reproductive hormones in baby rats (mIU/ml).** a: The level of T, b: The levels of E, FSH and LH. Results are presented as mean ± SE (n=15). *: significantly different from the control group (p<0.05), **: significantly different from the control group (p<0.01).

The effect of taurine on reproductive hormones in baby rats is presented in Fig. 1. The concentrations of T, E and LH were significantly increased when treated with taurine, but the concentration of FSH had no changes. The levels of T and LH were obviously decreased by β-alanine administration, but the levels of E and FSH showed no difference compared with the control group.

**Figure 2:**
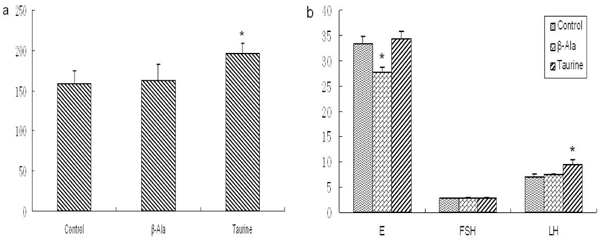
**The levels of reproductive hormones in adult rats (mIU/ml).** a: The level of T, b: The levels of E, FSH and LH. Results are presented as mean ± SE (n=10). *: significantly different from the control group (p<0.05), **: significantly different from the control group (p<0.01).

**Figure 3:**
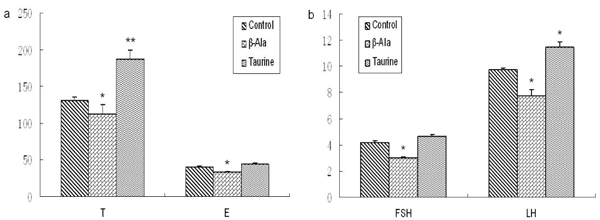
**The levels of reproductive hormones in aged rats (mIU/ml).** a: The level of T, b: The levels of E, FSH and LH. Results are presented as mean ± SE (n=10). *: significantly different from the control group (p<0.05), **: significantly different from the control group (p<0.01).

As Fig. 2 and Fig. 3 illustrates, the levels of T and LH were obviously increased by taurine administration in adult and aged rats, but the levels of E and FSH showed no significant changes. The level of E was significantly decreased by β-alanine treatment in adult rats, but the levels of T, FSH and LH had no changes. The aged rats treated by β-alanine had significant reductions in reproductive hormone.

### Testis biochemical indicator analysis

**Figure 4:**
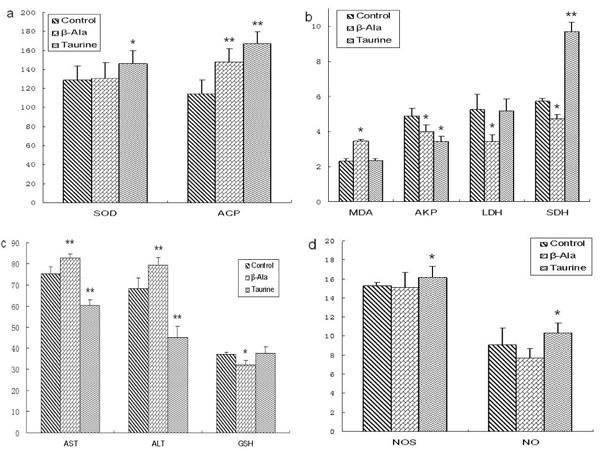
**The levels of testis biochemical indicators in adult rats.** a: the levels of SOD and ACP (U/mg prot), b: the levels of MDA (nmol/mg prot), AKP (U/mg prot), SDH (U/mg prot) and LDH (U/mg prot), c: the levels of AST (U/mg prot), ALT (U/mg prot) and GSH (μg/mg prot), d: the levels of NOS (U/mg prot) and NO (pmol/mg prot). Results are presented as mean ± SE (n=10). *: significantly different from the control group (p<0.05), **: significantly different from the control group (p<0.01).

As shown in Fig.4, the levels of SOD, ACP, SDH, NOS and NO were obviously increased by taurine administration in adult rats, the levels of AKP, AST and ALT were significantly decreased, but the levels of MDA, LDH and GSH had no changes. However, the levels of MDA, AST and ALT were significantly increased by β-alanine supplement in adult rats, the levels of AKP, LDH, SDH and GSH were obviously reduced, and the levels of SOD, NOS and NO had no changes.

**Figure 5:**
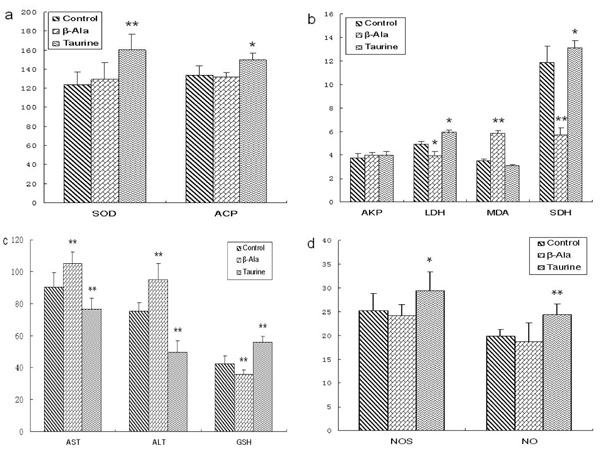
**The levels of testis biochemical indicators in aged rats.** a: the levels of SOD and ACP (U/mg prot), b: the levels of MDA (nmol/mg prot), AKP (U/mg prot), SDH (U/mg prot) and LDH (U/mg prot), c: the levels of AST (U/mg prot), ALT (U/mg prot) and GSH (μg/mg prot), d: the levels of NOS (U/mg prot) and NO (pmol/mg prot). Results are presented as mean ± SE (n=10). *: significantly different from the control group (p<0.05), **: significantly different from the control group (p<0.01).

The effect of taurine on the testis biochemical indicator in the aged rats was presented in Fig. 5. The levels of SOD, ACP, LDH, SDH, NOS, NO and GSH were significantly elevated by taurine administration in aged rats, the levels of AST and ALT were significantly decreased, but the levels of AKP and MDA had no changes. However, the levels of MDA, AST and ALT were obviously increased by β-alanine supplement in aged rats, the levels of LDH, SDH and GSH were significantly reduced, but the levels of SOD, AKP, NOS and NO had no changes.

### Sperm quality analysis

**Figure 6:**
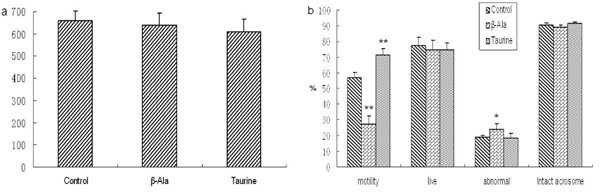
**The sperm quality of adult rats.** a: the numbers of spermatozoa, b: the rates of spermatozoa motility, live spermatozoa, abnormal and intact acrosome spermatozoa. Results are presented as mean ± SE (n=10). *: significantly different from the control group (p<0.05), **: significantly different from the control group (p<0.01).

**Figure 7:**
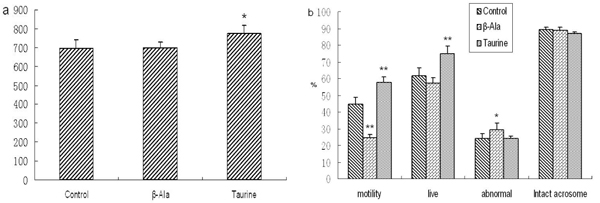
**The sperm quality of aged rats.** a: the numbers of spermatozoa, b: the rates of spermatozoa motility, live spermatozoa, abnormal and intact acrosome spermatozoa. Results are presented as mean ± SE (n=10). *: significantly different from the control group (p<0.05), **: significantly different from the control group (p<0.01).

As shown in Fig. 6 and Fig. 7, the motility of spermatozoa was obviously increased by taurine administration in adult rats, but there were no changes in other sperm quality. The numbers and motility of spermatozoa, and the rate of live spermatozoa were significantly increased by taurine supplement in aged rats, but there were no changes in the rates of intact acrosome and abnormal spermatozoa. The motility of spermatozoa was significantly decreased by β-alanine administration in adult and aged rats, but the rate of abnormal spermatozoa was obviously increased, the other sperm quality had no changes.

## Discussion

### Effects of taurine on reproductive hormones

It is well known that FSH, LH, T and E are the major hormones in male animals, which play important roles in male reproduction. In male animals, T and E are produced by testis. The secretion of T and E are regulated by FSH and LH which were produced by adenohypophysis.

In the present study, the effect of taurine on reproductive hormones was detected in male rats of different ages. The results showed that the levels of T and LH were obviously increased in rats of different ages, and the level of E was also significantly elevated in baby rats. However, β-alanine significantly lowered the concentrations of T and LH in baby rats, the concentration of E in adult rats, and the levels of four kinds of reproductive hormones in aged rats. The results were in agreement with the findings of Xiao et al. who suggested that the relative weight of the testis, and the levels of T and LH were obviously increased by taurine administration in broilers, and taurine had a promotive effect in the development of testis [24]. We have also previously identified that taurine can be biosynthesized by testis, and can stimulate the secretion of T in vivo and in vitro [18].

It has been reported that the secretion of LH had no changes by intraperitoneal injection of taurine [25]. The result is not similar to our observation about the effect of taurine on LH. The difference may be due to the concentration and the method of taurine administration.

### Effects of taurine on testis biochemical indicators

Testes are the most important organs in male reproduction, which are involved in the functions of spermatogenesis and testosterone secretion. In male animals, many kinds of enzymes are closely correlated to the functions of testis [26-29]. To evaluate the effect of taurine on testis biochemical metabolism, the levels of ACP, AKP, LDH, SDH, AST, ALT, SOD, MDA, NOS and NO were detected in testes of adult and aged rats.

In testis, AKP is associated with the division of spermatogenic cells and the transportation of glucose to spermatogenic cells. ACP is one of the markers of dyszoospermia that associated with the denaturation of seminiferous epithelium and phagocytosis of sertoli cells. In the present study, the activity of ACP was significantly increased by taurine supplementation in testis of adult and aged rats, but the activity of AKP was obviously decreased in testis of adult rats. In adult rats, the activity of ACP was obviously elevated by β-alanine administration in testis, and the activity of AKP was significantly decreased. The results indicated that taurine may be important in spermatogenesis by improving the lipid and energy metabolism, increasing the spermatogenic cells division in testis.

LDH and SDH are widely distributed and located in the seminiferous tubules and germ cells, which is associated with the maturation of spermatogenic cells, testis and spermatozoa and the energy metabolism of spermatozoa. Our results showed that the activity of SDH was significantly increased by taurine administration in adult rats, and the activities of SDH and LDH were obviously elevated in aged rats. However, the activities of SDH and LDH were significantly decreased by β-alanine administration in adult and aged rats. The results suggested that taurine plays an important role in the maturation and energy metabolism of spermatogenic cells and spermatozoa.

AST and ALT are the important aminotransferase and are widely distributed in mitochondrion, which is associated with the integrality of spermatozoa acrosome and cells stress. The activities of AST and ALT increased when the membrane of spermatozoa was damaged, and the rate of intact acrosome spermatozoa decreased. The present results showed that the activities of AST and ALT were obviously decreased by taurine supplement in testis of adult and aged rats, but were significantly increased by β-alanine supplement. The results indicated that taurine is an important factor that improves the ability of antinociception and anti-stress in testis cells including spermatogenic cells, and protects spermatozoa.

NO has been shown to be an important paracrine messenger and neurotransmitter that promotes homeostasis and various functions in many tissues. In the testis, NO and NOS are thought to regulate an array of functions, including sperm motility, maturation and germ cell apoptosis, Sertoli cell tight junction dynamics, and Leydig cell steroidogenesis [30,31]. The present results showed that the levels of NOS and NO were obviously increased by taurine administration in adult and aged rats, but had no changes after β-alanine administration. The results indicated that taurine may be essential to the function of testis.

SOD is a metalloprotein and accomplishes its antioxidant functions by enzymatically detoxifying the peroxides and superoxide anion. GSH is one of the most important compounds, which helps in the detoxification and excretion of oxygen radicals. MDA is one of several low-molecular-weight end products formed via the decomposition of certain primary and secondary lipid peroxidation products. In the present study, the levels of SOD, NOS and NO were significantly increased by taurine administration in adult and aged rats, and the activity of GSH was significantly increased in aged rats. The level of MDA was obviously increased by β-alanine supplement in experimental rats, but the activity of GSH was significantly decreased. The results indicated that taurine can improve the testis oxidative stability, inhibit lipid peroxidation, and promotes homeostasis of testis.

There were evidences that the levels of SDH, SOD, GSH, AST, ALT were significantly increased by a taurine supplement in serum, and the level of MDA decreased [32-35]. But it has been reported that the levels of NOS and NO obviously decreased in serum and many tissues [36,37], which was not in agreement with our results. It may be attributed to the difference of the detected tissues.

### Effects of taurine on the sperm quality

Taurine have been found in spermatozoa and seminal fluid of numerous species and are known to have beneficial effects on sperm characteristics in mammals [9,38]. It has been suggested that taurine play important roles in the maintenance and stimulation of sperm motility and stimulation of capacitation and acrosome reactions in vivo and in vitro [14,39]. It also has been found that taurine could inhibit lipid peroxidation in rabbit spermatozoa and protect the loss of motility [13]. In addition, taurine have been reported to improve either the initial post-thaw motility of ram sperm or the duration of motility of frozen–thawed ram sperm [40], and its cryoprotective effect may be attributable to its osmoregulation rather than to its antioxidant properties.

The present results showed that taurine could significantly increase the motility of sperm in adult rats, but has no obvious effects on the other semen quality. In aged rats, taurine could obviously increase the numbers and the motility of sperm, and the rate of live sperm, but has no significant effects on the rate of intact acrosome sperm. However, β-alanine could significantly decrease the motility of sperm, and increase the rate of abnormal sperm in adult and aged rats. The results indicated that taurine can improve the semen quality in male animals especially in aged male animals.

It has been found that aging resulted in a significant decline in serum and testis taurine content [19,21]. Our unpublished data also has identified that the biosynthesis of taurine in rat testis declined as aging occurred. The results of the present study demonstrated that male reproduction can be improved by taurine administration in aged rats. The improved effects of taurine on aged male reproduction may be attributed to the stimulation of T secretion, promotion of testis homeostasis, and antioxidation.

In summary, our results indicated that there are beneficial effects of taurine on male reproduction. The mechanisms of the beneficial effects are complex and unclear but may include the modulation of calcium levels, osmoregulation, membrane stabilization and antioxidation.

## Conclusion

In conclusion, the present study demonstrated that taurine supplement could stimulate the secretion of LH and T, increase the levels of testicular marker enzymes, elevate testicular antioxidation and improve sperm quality. The results implied that taurine plays important roles in male reproduction especially in aged animals, but the exact effect and mechanism need to be further investigated.

## List of abbreviations used

ACP: acid phosphatase; AKP: alkaline phosphatase; ALT: alanine aminotransferase; AST: aspartate aminotransferase; E: estradiol; FSH: follicle-stimulating hormone; GSH: reduced glutathione hormone; LDH: lactate dehydrogenase; LH: luteinizing hormone; MDA: malondialdehyde; NO: nitric oxide; NOS: nitric oxide synthase; SDH: sorbitol dehydrogenase; SOD: superoxide dismutase; T: testosterone; β-Ala: β-alanine

## Competing interests

The authors declare that they have no competing interests.

## Authors' contributions

Jiancheng Yang carried out the testis biochemical index studies, participated in the study design and drafted the manuscript. Gaofeng Wu carried out the hormones study. Qiufeng Lv participated in the sperm quality study. Shumei Lin participated in the design of the study and performed the statistical analysis. Jianmin Hu conceived of the study, and participated in its design and coordination and helped to draft the manuscript. All authors read and approved the final manuscript.
